# An Incidental Finding of a Sphenoid Sinolith: A Case Report

**DOI:** 10.7759/cureus.61386

**Published:** 2024-05-30

**Authors:** Alexandria Sobczak, Randy Felber, Lexie Leon, Alejandro Biglione

**Affiliations:** 1 Dr. Kiran C. Patel College of Osteopathic Medicine, Nova Southeastern University, Fort Lauderdale, USA; 2 Dr. Kiran C. Patel College of Osteopathic Medicine, Nova Southeastern University, Clearwater, USA; 3 Internal Medicine, Wellington Regional Medical Center, Wellington, USA

**Keywords:** sinolith, case report, calcification, sphenoid sinus, sinusitis

## Abstract

Sinoliths are mineral deposits that occur within the paranasal sinus due to long-standing obstruction and lack of drainage. It is a rare differential diagnosis for intrasinus lesions found on imaging. On computed tomography (CT) of the head, these calcifications are visualized as dense radiopaque bodies within the sinuses. Typically, patients with sinoliths are asymptomatic, but if complications of chronic obstruction and recurring sinusitis arise, endoscopic removal of the sinolith may be recommended. Here, we present a 95-year-old female found to have a sinolith in the sphenoid sinus on incidental imaging. This report discusses the etiology, pathophysiology, clinical presentation, radiologic findings, and management of sinoliths.

## Introduction

A sinolith is a calcified mass found within a paranasal sinus cavity. This is a rare occurrence that most commonly presents in the maxillary sinuses as an antrolith. It is less commonly found in the frontal, ethmoid, or sphenoid sinuses as a sinolith [1,2]. This is associated with chronic sinusitis including stagnant mucus and fungal balls. It can also develop from a foreign body [2,3]. Although the etiology is not entirely understood, sinolith formation is thought to be due to long-standing sinus obstruction and lack of drainage [1,4]. 

Intrasinus calcifications often go unreported as they are typically asymptomatic and found incidentally [5]. The most commonly reported symptoms for symptomatic calcifications include headache, dizziness, facial pain, and epistaxis [1,4]. 

Intrasinus calcifications can be identified on CT or MRI. If identified on imaging, an otolaryngologist should be consulted in cases of patients with symptoms or non-fungal causes to further investigate whether surgical removal would be beneficial for the patient in order to help prevent possible complications of prolonged obstruction [5]. 

In this report, we present the case of a 95-year-old female found to have a sinolith in the sphenoid sinus. This report illustrates the prevalence, etiology, pathophysiology, clinical presentation, radiographic findings, and management of a sinolith. 

## Case presentation

A 95-year-old female with a past medical history of chronic pain, transient ischemic attacks (TIA), atrial fibrillation, and chronic sinusitis presented to the emergency department (ED) via emergency medical services from a nursing home with complaints of lethargy, weakness, headache, anosmia, and mild facial tenderness around her nose and eyes for the past two days. A review of systems was significant for weakness, fatigue, nonproductive cough, nasal congestion, and insomnia. She denied epistaxis, fever, chest pain, shortness of breath, abdominal pain, nausea, diarrhea, dysuria, or urinary frequency. The patient denied alcohol, tobacco, or illicit drug use. The patient’s medications included rivaroxiban 20 mg, due to a history of multiple transient ischemic attacks and atrial fibrillation, aspirin 81 mg, duloxetine 60 mg, and tramadol 50 mg.

Initial vital signs included a temperature of 36.7 C, heart rate of 87 beats per minute (bpm), respiratory rate of 17 breaths per minute, blood pressure of 182/74 mmHg, oxygen saturation (SpO2) of 94% on room air, and a body mass index (BMI) of 19.49 kg/m^2^. On presentation, the patient was awake, lying comfortably in bed, in no acute cardiopulmonary distress, non-toxic, non-diaphoretic, and oriented to person, place, and time. On the physical exam, her heart rate and rhythm were normal. No wheezing, rales, or rhonchi were noted and the lungs were clear to auscultation bilaterally. Anterior rhinoscopy exam revealed diffuse nasal mucosal edema and secretions. An abnormal initial complete metabolic panel is included in Table 1 and Table 2. All lab values were based on this institution's range of normal and abnormal values. 

**Table 1:** Complete blood count laboratory values on admission

	Initial Value	Normal Range
Hemoglobin	13.6 gm/dL	14.0-18.0gm/dL
Hematocrit	38.9%	40.0-54.0%
Platelet Count	176 x10^3^/mcL	150-450 x 10^3^/mcL

**Table 2:** Complete metabolic panel laboratory values on admission

	Initial Value	Reference Range
Glucose	124 mg/dL	74-106 mg/dL
Sodium	130 mmol/L	135-148 mmol/L
Potassium	3.4 mmol/ L	3.6-5.2 mmol/L
Chloride	99 mmol/L	95-110 mmol/L
Bicarbonate	28 mEq/L	21-32 mEq/L
Blood urine nitrogen	15 mg/dL	7-18 mg/dL
Creatinine	0.54 mg/dL	0.55-1.02 mg/dL

**Figure 1:**
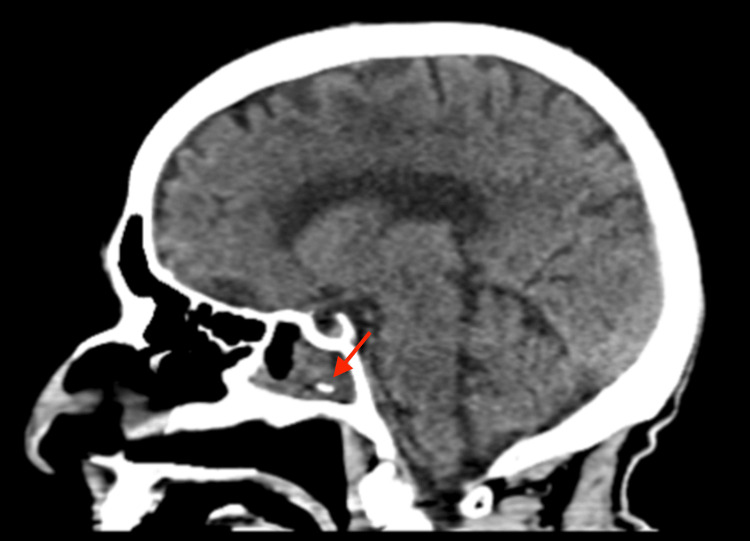
Head CT without contrast (sagittal view) showing calcification in the sphenoid sinus

**Figure 2:**
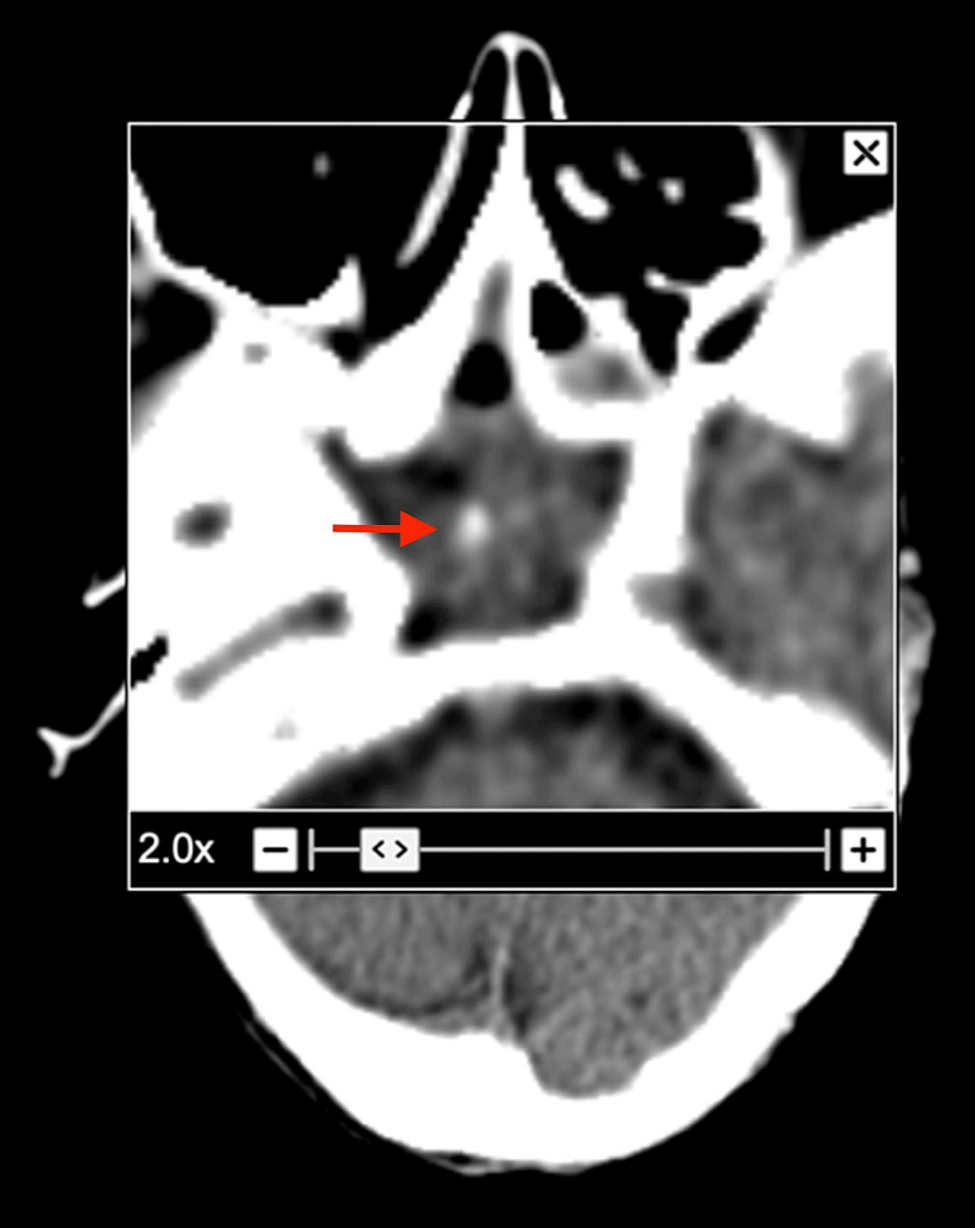
Head CT without contrast (axial view) with magnification showing calcification in the sphenoid sinus

In the emergency department, she also underwent a computerized tomography (CT) of the head and brain without contrast. While no hemorrhage, edema, or infarct was found in the brain, the study showed marked mucosal thickening of the sphenoid sinus and a hyperdense oval soft tissue density with calcification was seen in the sphenoid sinus (Figure 1 and Figure 2). A chest x-ray was also performed and no infiltrates, effusion, or pneumothorax was visualized. The patient was admitted to the internal medicine floor for further evaluation and was found positive for COVID-19. She was started on intravenous fluids and nirmatrelvir:ritonavir 300 mg:100 mg.

Throughout her two days in the hospital, the patient improved, and she was cleared for discharge. She was instructed to take Paxlovid (nirmatrelvir/ritonavir) twice daily for two more days to complete the course. She was also prescribed fluticasone 50 mcg in each nostril once daily to relieve her symptoms of sinusitis. The patient was instructed to follow up with her primary care physician after discharge. The patient did not want to undergo endoscopic removal of the calcification due to her age and lack of symptoms. She was advised to follow up with an otolaryngologist and repeat imaging in six months.

## Discussion

Sinolith formation is a unique and uncommon complication of chronic sinus obstruction. When these intrasinus calcifications occur, they often go unreported, as many individuals are asymptomatic. When symptoms arise, patients most commonly present with headache, but may also present with epistaxis, postnasal drip, or facial pain [2,4].

In sinolith formation, lack of sinus clearance and continuous sinus obstruction play pivotal roles in calcium deposition. Sinus obstruction, chronic infection, and poor drainage allow minerals to deposit within the sinuses leading to the formation of calcification overtime. Etiologies of sinolith formation include chronic trauma, mucosal plugging, and fungal infections [4,6]. Exogenous obstructions of the sinuses include stone, cotton, and paper. Blood clots, mucosal necrosis, and tooth fragments are common endogenous sources of sinolith formation [1,4]. 

While the etiology and pathogenesis of sinolith formation are still not well understood, inflammatory diseases such as chronic sinusitis may predispose to sinolith formation because the combination of increased inflammatory processes and lack of mucosal clearance can lead to calcium deposition [2]. Although our patient had a history significant for chronic sinusitis, it was hard to tell which condition developed first. While chronic sinusitis is thought to be a risk factor for sinolith formation, the inability to drain the sinus due to mineral deposits can also lead to recurring sinus infections. Although causation was unknown in this clinical case, the patient’s sinolith may have been associated with her history of chronic inflammatory disease. 

Since sinoliths are asymptomatic in the majority of people, they are not typically visualized or suspected until they are discovered incidentally on imaging [7]. Similarly, this patient’s sphenoid sinus calcification was discovered after undergoing a head CT to rule out ischemic or hemorrhagic stroke. CT is the gold standard for visualizing the paranasal sinuses and helps make the diagnosis of a sinolith [2]. On CT, these calcifications can be identified as dense, irregular, well-defined radiopaque masses within the sinus [6,8]. Sinoliths may present with a rough or smooth border and may be oval, round, or irregular in shape [1]. They are most commonly found along the maxillary sinus, where they are referred to as antroliths, but have also been visualized in the frontal, ethmoid, or sphenoid sinuses [1]. Some differential diagnoses for these bony densities include an osteoma and calcified mucus retention. Osteomas most commonly occur in the frontal sinus and due to their tumoral growth pattern, can be seen characteristically attached to the origin bone via a pedicle. [9]. The high density of this calcification on CT makes mucus retention unlikely [9]. An isolated lesion within the sinus is usually due to a long-standing infection [2]. In the current case, because the calcified mass was an isolated calcification unattached to the bone, sinolith was the most likely diagnosis. While endoscopic removal and histopathology would further confirm this diagnosis, the patient refused due to her age and lack of symptoms. 

In patients who are asymptomatic and without complications, surgery is not always indicated. However, if complications or symptoms are present or if there is concern for ruling out alternative diagnoses, surgery may be recommended [3]. If a sinolith is causing recurring sinusitis or fungal infection, surgical intervention may be required [5]. Endoscopic removal of the sinus stone is a first-line surgical treatment used when indicated. Endoscopic removal, while minimally invasive, may provide further information regarding the sinolith such as color and composition [4]. In the case of our patient who refused endoscopic removal, follow-up with an otolaryngologist and repeat imaging was recommended.

## Conclusions

Intrasinus calcification is a process that occurs with long-standing exogenous or endogenous sinus obstruction. Sinoliths should be considered in patients with chronic sinusitis and can appear as dense radiopaque masses on CT and MRI. Although rare and typically asymptomatic, sinoliths may carry complications such as chronic sinusitis and infection. Therefore, it is important to keep them as a differential diagnosis and further investigate whether surgical removal would be beneficial for the patient.
